# The prevalence of neonatal jaundice and risk factors in healthy term neonates at National District Hospital in Bloemfontein

**DOI:** 10.4102/phcfm.v10i1.1582

**Published:** 2018-04-12

**Authors:** Hanneke Brits, Jeanie Adendorff, Dyanti Huisamen, Dahne Beukes, Kristian Botha, Hanre Herbst, Gina Joubert

**Affiliations:** 1 Department of Family Medicine (G19), University of the Free State, South Africa; 2 School of Medicine, University of the Free State, South Africa; 3 Department of Biostatistics (G31), University of the Free State, South Africa

## Abstract

**Background:**

Neonatal jaundice affects one in two infants globally. The jaundice is the result of an accumulation of bilirubin as foetal haemoglobin is metabolised by the immature liver. High serum levels of bilirubin result in lethargy, poor feeding and kernicterus of the infant.

**Aim:**

The main aim of this article was to determine the prevalence of neonatal jaundice and secondly to explore its risk factors in healthy term neonates.

**Setting:**

Maternity ward, National District Hospital, Bloemfontein, South Africa.

**Methods:**

In this cross-sectional study, mothers and infants were conveniently sampled after delivery and before discharge. The mothers were interviewed and their case records were reviewed for risk factors for neonatal jaundice and the clinical appearance and bilirubin levels of the infants were measured with a non-invasive transcutaneous bilirubin meter.

**Results:**

A total of 96 mother-infant pairs were included in the study. The prevalence of neonatal jaundice was 55.2%; however, only 10% of black babies who were diagnosed with jaundice appeared clinically jaundiced. Normal vaginal delivery was the only risk factor associated with neonatal jaundice. Black race and maternal smoking were not protective against neonatal jaundice as in some other studies.

**Conclusion:**

More than half (55.2%) of healthy term neonates developed neonatal jaundice. As it is difficult to clinically diagnose neonatal jaundice in darker pigmented babies, it is recommended that the bilirubin level of all babies should be checked with a non-invasive bilirubin meter before discharge from hospital or maternity unit as well as during the first clinic visit on day 3 after birth.

## Introduction

The term ‘jaundice’ is used to describe the yellow-orange discoloration of the skin and sclera because of excessive bilirubin in the skin and mucous membranes.^1,2^ Jaundice itself is not a disease but rather a symptom or sign of a disease. Bilirubin is mainly formed when the haem component of red blood cells are broken down in the spleen to biliverdin and then unconjugated bilirubin.^3^ As bilirubin is not water soluble, it is transferred via the bloodstream from the spleen to the liver, bound to the plasma protein albumin. In this form, it is known as conjugated bilirubin, which is then secreted into the gall. In the gut it is further metabolised to other gall pigments and then excreted in the faeces.^3^

The mechanism of neonatal jaundice is the imbalance between bilirubin production and conjugation, which results in increased bilirubin levels.^4^ This imbalance is mainly because of the immature liver of the neonate and the rapid breakdown of red blood cells, which may be multifactorial.^3,4,5,6^ At bilirubin levels of between 85 µmol/L and 120 µmol/L, neonatal jaundice can be diagnosed clinically.^7,8,9^ Kramer described the difficulty of clinically diagnosing neonatal jaundice in darker pigmented neonates.^9^ A study by Moyer et al. found that the clinical diagnosis of neonatal jaundice is ‘neither reliable nor accurate’.^10^ Neonatal jaundice is very common and is present in 60% of term babies and up to 80% of premature babies.^4,5,6,8^ The main risk factors identified for neonatal jaundice include prematurity and neonatal sepsis.^8,11,12,13,14^ In physiological jaundice, it is only the unconjugated bilirubin levels that are raised, because of immaturity of the liver in the absence of any other illness. In pathological jaundice, there are underlying conditions that either increase the production of bilirubin or decrease the excretion. In order to treat pathological jaundice, the underlying conditions must be treated.^4^

Neonatal jaundice is usually not harmful and a self-limiting condition; however, very high levels of bilirubin may cause permanent brain damage, a condition called kernicterus.^1^ Therefore, it is important to diagnose neonatal jaundice and manage it appropriately.

**FIGURE 1:**
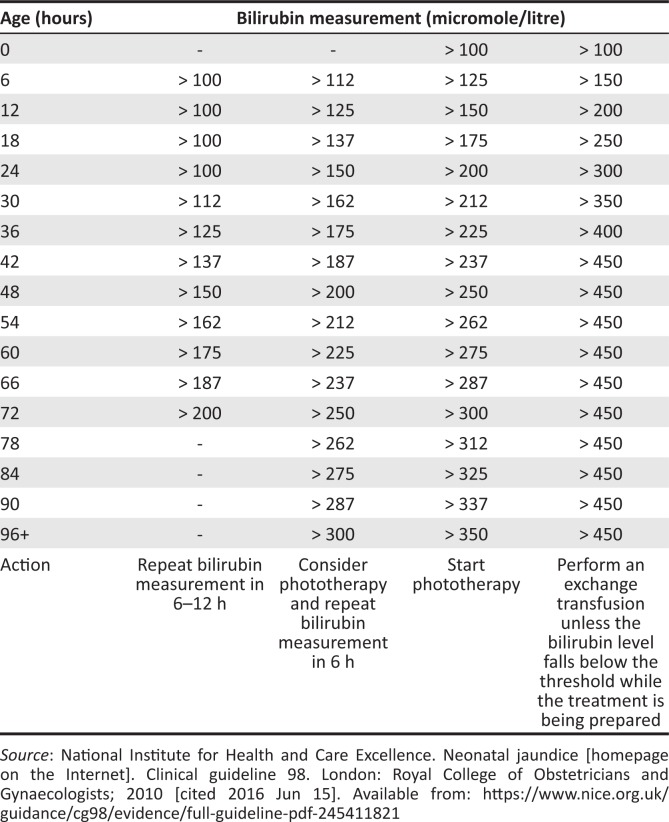
Threshold table: Consensus-based bilirubin thresholds for management of babies 38 weeks or more gestational age with hyperbilirubinaemia.^1^

A guideline, compiled by the heads of neonatal departments at South African medical schools, with management principles for term babies with neonatal jaundice at primary health care was published in 2006.^15^ According to this and other guidelines, the management of neonatal jaundice can be done through observation, phototherapy or exchange transfusion, according to the bilirubin levels and the age of the neonate.^1,5,15^ The current guideline stipulated in the Standard Treatment Guidelines and Essential Medicines List (STG and EML) is to use weight and gestational age to decide on treatment, which may cause confusion if it does not correlate or if the baby loses weight.^16^ The National Institute for Health and Clinical Excellence (NICE) Clinical Guideline 98^1^ provides the following management guidelines for neonatal jaundice:

- Provide information to all parents and caregivers of neonates regarding neonatal jaundice.
- Examine all babies and identify risk factors for neonatal jaundice.
- Inspect the skin, sclera and gums of the naked baby, in natural light, for the presence of jaundice.
- Measure the bilirubin in all babies with clinical jaundice with a non-invasive, transcutaneous bilirubin meter.
- Use the threshold table (Figure 1)^1^ to decide on further management.

The NICE guideline is much easier to use than the STG guideline as it only takes into consideration the infants’ age and not their weight. According to the threshold table and the NICE guideline,^1^ the management is then divided into the following categories:

- If value is lower than threshold, render normal neonatal care.
- If value is in first bilirubin column, repeat the bilirubin measurement within 6–12 h.
- If value is in second bilirubin column, consider phototherapy and repeat the bilirubin measurement in 6 h.
- If value is in third bilirubin column, start phototherapy.
- If value is in last bilirubin column, start phototherapy and arrange for exchange transfusion.
- If the baby develops jaundice within 24 h of birth or any sign of sepsis is present, the baby must be managed or referred urgently for specialist care.

The use of a transcutaneous bilirubin meter is widely advocated because of its almost 100% accuracy to detect hyperbilirubinaemia in term infants, safety owing to non-invasiveness, minimal operational costs and ability to be used in darker pigmented neonates.^1,17^ The NICE guideline also reported on the cost-effectiveness of transcutaneous bilirubin meters. It was calculated that the prevention of one or two cases of kernicterus per year justifies the cost of a transcutaneous bilirubin meter.^1^

### Aim

The aims of the study were to determine the prevalence of neonatal jaundice and to explore its risk factors in healthy term neonates at National District Hospital in Bloemfontein, South Africa.

## Research methods and design

### Study design and setting

This study was a cross-sectional study.

National District Hospital is an academic district hospital with 23 maternity beds, located between the secondary and tertiary academic hospitals in Bloemfontein. A total of 2402 neonates are born in the hospital annually and the caesarean section (C-section) rate is 31.85%. It is a training hospital and most elective C-sections from the area as well as the southern Free State are referred to this hospital. Because of transport issues, some healthy patients stay in the hospital for a few days after discharge, especially over weekends. Mainly uncomplicated cases are managed in the hospital. All mothers and infants with complications are transferred to the nearby secondary hospital. Before the study, bilirubin levels were only measured if an infant appeared clinically jaundiced. If a baby is diagnosed with neonatal jaundice but otherwise healthy, the baby is kept in the hospital for phototherapy.

### Sample population and sampling strategy

The study population included all healthy term neonates born at National District Hospital in Bloemfontein, between 01 August and 31 December 2016. Convenience sampling was used by including all newborn babies who met the inclusion criteria and who were in hospital on the days that the student researchers visited the hospital. These visits were dependent on classes and training schedules, as well as tests and examinations. The student researchers visited the hospital 38 times at regular intervals during the week and on weekends to limit bias.

Inclusion criteria for the study were the following:

- term babies defined as neonates born after 37 completed weeks of gestation
- healthy babies not on any medication, except Nevirapine for the prevention of mother-to-child transmission of HIV
- neonates 6 h and older
- mothers 18 years and older
- mothers who gave informed consent.

### Data collection

After written informed consent was obtained from the mothers, demographic data of mothers and babies were collected on a data collection form, together with the measured bilirubin levels of the babies. Data were obtained from patient files and structured interviews with the mothers. None of the mothers refused to provide consent. The bilirubin level was measured using a Bilicheck^®^ meter, which is a non-invasive electronic meter that measures bilirubin levels when pressed against the baby’s skin. The table from the NICE guidelines^1^ was then used to determine if the baby had jaundice and what treatment the baby should receive. The cut-off values varied between 100 µmol/L and 200 µmol/L depending on the age of the neonate.

#### Pilot study

A pilot study was conducted on 10 mother-baby pairs to test the data collection form and to ensure that the student researchers were familiar with the Bilicheck^®^ meter and guidelines. No major changes were made to the data collection form and the 10 cases were included in the main study.

### Data analysis

Data were transferred to an Excel data sheet and checked by two student researchers for accuracy. Data analysis was done by the Department of Biostatistics, Faculty of Health Sciences, University of the Free State (UFS), using SAS version 9. Results were summarised by frequencies and percentages (categorical variables) and means, standard deviations or percentiles (numerical variables, based on data distribution). Associations between risk factors and neonatal jaundice were assessed using chi-squared or Fisher’s exact tests.

### Ethical considerations

The Health Sciences Research Ethics Committee of the UFS as well as the Free State Department of Health approved the study (HSREC-S 33/2016). Ministerial approval for non-therapeutic research on minors was also granted; however, during the study some babies were identified who received therapeutic interventions.

Participation in the study was voluntary. All mothers, 18 years and older, gave informed consent for the use of their hospital file, an interview and the non-invasive measurement of the baby’s bilirubin level.

## Results

A total of 96 mother-infant pairs were included in the study. As per inclusion criteria, all babies were term and healthy. The age of the mothers varied between 18 and 36 years, with a mean age of 26.5 years. The mean weight of the babies was 3.15 kg, ranging from 2.1 kg to 4.39 kg, and the mean gestation was 38.5 weeks.

### Prevalence of neonatal jaundice

**FIGURE 2:**
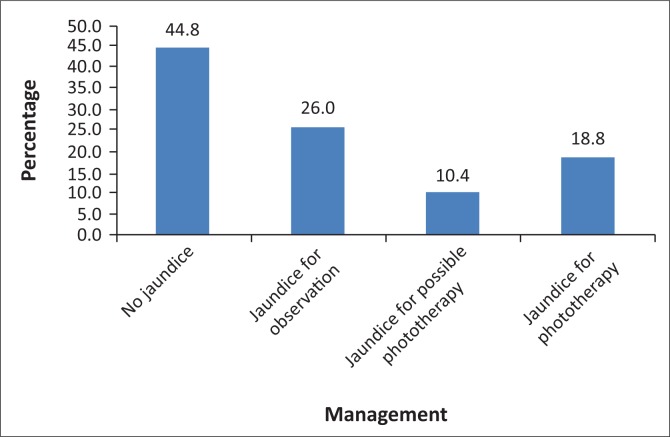
Percentage of neonates per management category (*n* = 96).

The prevalence of neonatal jaundice, using the NICE guideline cut-off values, was 55.2% (*n* = 53). In Figure 2, the percentage of neonates per management category is depicted. No neonate needed an exchange transfusion. Only nine (17%) of the 53 infants diagnosed with jaundice appeared clinically jaundiced, of whom four were black infants.

Different risk factors were assessed for possible association with neonatal jaundice.

### Demographic risk factors

All the patients were asked to classify themselves according to race. The majority of patients classified themselves as black people. Table 1 summarises the race distribution and the presence or absence of neonatal jaundice. No statistically significant difference between the race groups was found (*p* = 0.60).

**TABLE 1:** The race distribution of the mothers, as classified by themselves, and the presence or absence of neonatal jaundice.

Race of mother	Neonatal jaundice		Total *n* (%)
	Present *n* (%)	Absent *n* (%)	
Black	41 (55.4)	33 (44.6)	74 (77.1)
Mixed race	8 (50.0)	8 (50.0)	16 (16.7)
White	3 (60.0)	2 (40.0)	5 (5.2)
Asian	1 (100.0)	0 (0.0)	1 (1.0)
**Total**	**53 (55.2)**	**43 (44.8)**	**96 (100.0)**

The age of the neonates at the time the bilirubin level was measured varied between 12 and 216 h, with a median age of 45 h. In neonates 72 h and older, the chance of neonatal jaundice was statistically significantly higher (*p* = 0.016). Of the neonates, 16% were in this group and 93.3% of them had neonatal jaundice. In Table 2, the indicated treatment is displayed per age group.

**TABLE 2:** The number of neonates per age category, with jaundice for observation, with jaundice for phototherapy or possible phototherapy, and without jaundice.

Age category	Jaundice present (*n* = 53)		Jaundice absent (*n* = 43) *n* (%)	Total (*n* = 96) *n* (%)
	For observation (*n* = 25) *n* (%)	For phototherapy or possible phototherapy (*n* = 28) *n* (%)		
< 24 h	7 (29.2)	4 (16.7)†	13 (54.2)	24 (25.0)
< 48 h	7 (17.1)	10 (24.4)	24 (58.5)	41 (42.7)
< 72 h	5 (31.3)	6 (37.5)	5 (31.3)	16 (16.7)
< 96 h	3 (60.0)	2 (40.0)	0 (0.0)	5 (5.2)
≥ 96 h	3 (30.0)	6 (60.0)	1 (10.0)	10 (10.4)

† , four babies were diagnosed with neonatal jaundice within 24 h after birth and needed further investigations. They were all in the ‘possible’ group and no underlying conditions were found.

### Antenatal risk factors

The majority of patients did not smoke (88.5%) and did not use alcohol (94.8%) or oral contraception (94.8%) during the pregnancy. None of these factors was statistically significantly associated with neonatal jaundice, but jaundice occurred more frequently in smokers (9/11, 81.8%) than in non-smokers (44/85, 51.8%), *p* = 0.10.

### Labour risk factors

Oxytocin was used in 19.8% of the deliveries to induce or augment the labour. The use of oxytocin was not associated with a higher incidence of neonatal jaundice (*p* = 0.44). Table 3 presents the mode of delivery. The majority of babies included in the study were born via C-section (64.6%). Normal vaginal delivery was associated with statistically more neonatal jaundice (*p* = 0.04).

**TABLE 3:** Mode of delivery and the presence or absence of neonatal jaundice.

Mode of delivery	Neonatal jaundice		Total (*n* = 96) *n* (%)
	Present (*n* = 53) *n* (%)	Absent (*n* = 43) *n* (%)	
Caesarean section (C-section)	29 (46.8)	33 (53.2)	62 (64.6)
Instrumental	1 (100.0)	0 (0.0)	1 (1.0)
Normal vaginal delivery	23 (69.7)	10 (30.3)	33 (34.4)

Ninety-five per cent of the mothers breastfed their babies exclusively, of which 56% of babies had jaundice. Of the five who did not breastfeed, two babies developed neonatal jaundice (*p* = 0.65).

## Discussion

The baseline characteristics regarding age (26.5 years vs. 25 years) and race (black 77.1% vs. 80%) of the mothers, as well as the mean birth weight (3.25 kg vs. 3.07 kg) of the neonates, compared well with that of national statistics for mothers attending and delivering at public health facilities in South Africa.^18,19,20^

Babies 72 h and older had a statistically significantly higher chance to be diagnosed with neonatal jaundice. This is in accordance with the natural progression of physiological jaundice, which usually peaks between days 3 and 5 after birth and then bilirubin levels return to normal by day 10.^1,7,8^ It is, however, worrisome that the majority of babies will not be in hospital after 72 h, where the jaundice can be diagnosed. After the study, a healthy baby clinic was started at the hospital where the bilirubin levels of all infants are measured between days 3 and 5.

The prevalence of neonatal jaundice was 55.2%, which is in accordance with the literature and guidelines where the prevalence is quoted to be between 50% and 60% for healthy term neonates.^1,6,7,8^ Of the babies, 19% qualified for phototherapy and a further 10.4% possibly qualified for phototherapy according to this study. The majority of babies who qualified for possible phototherapy received phototherapy, mainly because of the difficulty to return for follow-ups. However, the number of babies who were admitted for phototherapy prior to this study was much lower, possibly because they were not diagnosed. A positive finding was that none of the healthy term neonates needed an exchange transfusion and was also not at risk for the development of kernicterus.

Risk factors identified in different studies for the development of neonatal jaundice in healthy term babies include Asian race, instrumental delivery, babies born via C-section, normal vaginal delivery, infant bruising, induction of labour with oxytocin and exclusive breastfeeding, while moderate smoking of the mother and black race may be protective.^8,11,12,13,14^ Although black race was described in these studies as protective against neonatal jaundice, it was not found in this study. In this study, the bilirubin levels of all the babies, and not only those who clinically appeared jaundiced, were measured as prescribed in the NICE guidelines.^1^ Only one Asian baby and five white babies were included in the study; therefore, it was not possible to make any conclusions regarding other races.

A total of 11% of mothers indicated that they smoked during pregnancy. Smoking did not have a protective effect against neonatal jaundice in this study population, although moderate smoking was associated with a statistically significant lower chance of neonatal jaundice in other studies.^11,14^

The high percentage of babies born via C-section (65% compared with the C-section rate of 32% for the hospital) in this study is possibly because of the fact that these babies usually stay 3 days in the hospital compared to babies born via normal vaginal delivery, who are usually discharged within 24 h after birth. The likelihood to be included in the study was therefore higher for babies born via C-section. Normal vaginal delivery was associated with a higher chance of neonatal jaundice. This is also found in a study performed in Iran, but the opposite of what was found in a study in West Bengal.^13,21^ A possible reason for the lower prevalence of neonatal jaundice in the C-section group is that the majority of C-sections were elective procedures performed at 39 weeks gestation and therefore the babies were not exposed to normal birth trauma and bruising. Only one baby was born by a vacuum extraction.

Breastfeeding was associated with neonatal jaundice in some studies, but most of them also linked the breastfeeding with low calorie intake and dehydration.^6,8,14^ As 95% of mothers breastfed in this study, the association with a higher prevalence of neonatal jaundice could not be investigated adequately.

### Study limitations

A limitation of the study is that convenience sampling was used, which contributed to a small study sample and the high percentage of babies born via C-section. The low numbers of participants with certain risk factors (e.g. smoking or alcohol use) made it difficult to investigate associations. Data on the gravidity of the mothers and the gender of the babies were not collected, which were identified as risk factors in some studies.^8,12,14^

## Conclusion

The prevalence of neonatal jaundice in healthy term babies at National District Hospital in Bloemfontein was 55.2%. Although 52% of sampled infants had jaundice on the Bilicheck^®^ meter, only 17% appeared clinically jaundiced. The consequence of a missed diagnosis and delayed treatment may cause serious morbidity (kernicterus). The Bilicheck^®^ meter is reliable, non-invasive, easy to use and cost-effective and should be available in all maternity units and clinics for screening of all infants before discharge and again on day 3. Although babies 72 h and older had a greater chance of neonatal jaundice, it cannot be considered as a risk factor, as it is in accordance with the normal course for the development of neonatal jaundice. The only risk factor identified in this study that could contribute to neonatal jaundice was normal vaginal delivery.

### Recommendations

Because of the fact that it is difficult to clinically diagnose neonatal jaundice in darker pigmented babies, it is recommended that the bilirubin level of all babies should be checked with a non-invasive bilirubin meter before discharge from hospital or maternity unit.

The bilirubin levels of all neonates should be measured again during the first clinic visit, preferably on day 3 or 4 after birth.
